# A Comparative Study of a New Retractor‐Assisted WILTSE TLIF, MIS‐TLIF, and Traditional PLIF for Treatment of Single‐Level Lumbar Degenerative Diseases

**DOI:** 10.1111/os.13289

**Published:** 2022-05-23

**Authors:** Huanan Liu, Jiaqi Li, Yapeng Sun, Xianzheng Wang, WeiJian Wang, Lei Guo, Fei Zhang, Peng Zhang, Wei Zhang

**Affiliations:** ^1^ Department of Spinal Surgery The Third Hospital of Hebei Medical University Shijiazhuang China

**Keywords:** lumbar degenerative disease, minimally invasive, single level, Wiltse approach

## Abstract

**Objectives:**

To compare the clinical efficacy of a new retractor‐assisted Wiltse transforaminal lumbar interbody fusion (TLIF), minimally invasive TLIF (MIS‐TLIF), and traditional posterior lumbar interbody fusion (PLIF) in treating single‐level lumbar degenerative diseases.

**Methods:**

A retrospective study was conducted by analyzing the clinical and imaging data of consecutive patients with single‐level lumbar degenerative diseases who underwent the new retractor‐assisted Wiltse TLIF, MIS‐TLIF, or traditional PLIF. This study enrolled 87 concurrent patients between June 2016 and December 2019 (Wiltse TLIF 29 cases; MIS‐TLIF 28 cases; PLIF 30 cases). The three groups were compared for perioperative indicators (including intraoperative blood loss, postoperative drainage volume, operation time, intraoperative fluoroscopy time, bedridden time), creatine kinase (CK), visual analog score (VAS), Oswestry disability index (ODI), Japanese Orthopaedic Association (JOA) score, intervertebral fusion rate, muscle atrophy, and fatty infiltration (including ratio of multifidus atrophy and ratio of lean‐to‐total cross‐sectional area [CSA]).

**Results:**

Intraoperative blood loss (*F* = 62.628, *p* < 0.001), postoperative drainage volume (*F* = 72.048, *p* < 0.001), and bedridden time (χ^2^ = 62.289, *p* < 0.001) were significantly lower in the MIS‐TLIF and Wiltse groups than in the PLIF group. The operative and intraoperative radiation times of the MIS‐TLIF group were significantly longer than those of the Wiltse and PLIF groups. The CK concentration in the Wiltse and MIS‐TLIF groups were significantly lower than those in the PLIF group 1 day (*F* = 9.331, *p* < 0.001) and 3 days after surgery (*F* = 15.967, *p* < 0.001). The PLIF group's back pain VAS score was higher than those of the Wiltse and MIS‐TLIF groups. The PLIF group had a higher ODI 6 months (*F* = 3.282, *p* = 0.042) and 12 months (*F* = 5.316, *p* = 0.007) after surgery and a lower JOA score than the Wiltse and MIS‐TLIF groups 6 months (*F* = 3.234, *p* = 0.044) and 12 months (*F* = 3.874, *p* = 0.025) after surgery. The ratio of multifidus atrophy in the PLIF group (41.70 ± 8.84%) was significantly higher than those of the Wiltse group (24.13 ± 6.82%) and the MIS‐TLIF group (22.35 ± 5.03%). The ratio of lean‐to‐total CSA in the PLIF group was lower than those of the Wiltse and MIS‐TLIF groups after surgery (*F* = 8.852, *p* < 0.001). MIS‐TLIF group showed longer operation time (169.11 ± 29.38 min) and intraoperative fluoroscopy time (87.61 ± 3.13 s) than the Wiltse group.

**Conclusion:**

Wiltse TLIF assisted by the new retractor is a more convenient and minimally invasive surgical method than the traditional PLIF and MIS‐TLIF methods, which are linked to a long learning curve and long operation and fluoroscopy time.

## Graphical abstract

Wiltse approach TLIF and MIS‐TILF reach the surgical site through the natural space between the multifidus and the longus muscle, reducing blood loss, bedtime, muscles atrophy, and low back pain. Compared with MIS‐TLIF, which requires long learning curve, long operation and fluoroscopy times, Wiltse TLIF assisted with a new type of retractor is more convenient and simpler.

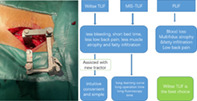

## Introduction

Lumbar fusion can be accomplished in a number of ways. Traditional open posterior surgery has become a commonly used surgical method for lumbar spine surgery due to its short learning period, sufficient decompression, wide applicability, and reliable operation outcome. However, due to the large incision and extensive paravertebral muscle dissection, the innervation and blood supply of the multifidus muscle are considerably damaged during the posterior lumbar surgery. Many patients (~25%–35%) have intractable low back pain (LBP) after surgery, accounting for an increase in back pain visual analog scale (VAS) scores by 3–5, which seriously affects the quality of life of the patients^1^. Therefore, reducing the incidence of complications, including soft tissue injury and LBP caused by surgery, has become the focus of many surgeons.

With the development of minimally invasive spine surgery to reduce injury, surgeons can choose techniques such as minimally invasive transforaminal lumbar interbody fusion (MIS‐TLIF), Wiltse TLIF, eXtreme lateral body interfusion (XLIF), and anterior lumbar interbody fusion (ALIF) according to the patient's condition. Lumbar surgery using the Wiltse approach can significantly reduce damage to muscles and nerves. In 1968, Wiltse first described the paraspinal sacrospinalis‐splitting approach to the lumbar spine. This approach was initially developed for the fusion of spondylolisthesis. It allowed the surgeon to approach the fusion area without cutting many of the supporting structures^2^. In 1988, Wiltse further described a posterolateral approach through the space between the multifidus and the longissimus to the foramina for the treatment of far lateral disc herniation, spinal canal stenosis, and lumbar spondylolisthesis^3^.

Because the Wiltse approach significantly reduces paraspinal muscle injury and blood loss, and direct exposure to the facet joint facilitates pedicle screw implantation, it has been further widely used in the treatment of thoracolumbar fracture without nerve injury. Foley *et al*. first published a report of minimally invasive transforaminal fusion in 2002, with an average operative time of 240 min and an estimated average blood loss of 75 ml^4^. Since its introduction, the MIS‐TLIF has demonstrated minimal soft tissue disruption and minimal destabilization of the spinal segment(s), leaving the smallest operative footprint possible while achieving the operative goal^5^. Wiltse TLIF and MIS‐TLIF are both minimally invasive surgical methods with the advantages of less bleeding, slight muscle injury, shorter hospital stay, and significantly reduced long‐term complications such as stubborn LBP. Therefore, they provide more possibilities for reducing surgical injuries and complications in patients^6^, ^7^, ^8^.

Numerous studies have reported clinical advantages of the Wiltse approach and MIS‐TLIF in treating degenerative lumbar diseases (blood loss 50–180 ml, hospital stay 2–5 days)^9^, ^10^. However, MIS‐TLIF still has the limitations of long fluoroscopy time and a long learning curve and causes tissue trauma to some extent due to the specific tubular compression to the muscle during the operation^5^, ^11^. In contrast, the Wiltse approach has the advantages of relatively short operation and fluoroscopy time and a smooth learning curve in its clinical application while reducing muscle injury. However, few studies have compared the MIS‐TLIF and Wiltse TLIF with conventional posterior lumbar interbody fusion (PLIF) in detail.

In addition, the traditional Wiltse TLIF approach requires an assistant to use a laminectomy retractor for continuous traction to expose the surgical field. To further increase the surgical convenience of the Wiltse approach, we independently designed a new retractor to assist field exposure. The purpose of this study was to: (i) introduce a new type of retractor for Wiltse TLIF, which is a simple, convenient device to improve exposure; (ii) compare the efficacy of this retractor‐assisted Wiltse TLIF with that of MIS‐TLIF and traditional PLIF to identify their advantages and disadvantages; and (iii) investigate the muscle injury including atrophy and fatty infiltration of the paraspinal muscle using quantitative MRI measurements.

## Materials and Methods

### Inclusion and Exclusion Criteria

The inclusion criteria were as follows: (i) a diagnosis of lumbar degenerative disease, including lumbar disc herniation with intervertebral instability, lumbar spinal stenosis, lumbar spondylolisthesis; (ii) with unilateral or bilateral lower limb symptoms (intermittent claudication or sciatica). After 3 months of conservative treatment, no obvious symptom relief was observed; and (iii) physical and imaging examinations confirmed single‐level lumbar disease.

The exclusion criteria were as follows: (i) lumbar spondylolisthesis ≥2° or accompanied by obvious spondylolysis; (ii) previous history of lumbar surgery; (iii) operative‐level infection, tumor, or fracture; (iv) combined with other severe cardiovascular and cerebrovascular diseases or other surgical contraindications.

### Baseline Clinical Data

A total of 87 consecutive patients with lumbar degenerative diseases who had undergone Wiltse TLIF (29 cases), MIS‐TLIF (28 cases), or PLIF (30 cases) in our department between June 2016 and December 2019 were included in this study. There were 35 patients (44.9%) with unilateral lower limb pain, 29 patients (32.2%) with lower limb pain and numbness, 20 patients (23.0%) with bilateral symptoms, and three patients (3.4%) with simple LBP. Twenty‐eight patients (35.9%) presented unilateral or bilateral lower limb muscle strength decline to varying degrees. Cauda equina syndrome was present in four patients (4.6%).

All the patients underwent lumbar radiography of the anterior and lateral positions, hyperextension and hyperflexion position, lumbar intervertebral disc CT, and lumbar MRI before surgery. Subsequently, 15 patients were found to have lumbar spondylolisthesis, and 21 patients had lumbar instability. All patients' physical examination results were consistent with their imaging changes, and all patients were confirmed to have single‐level degenerative diseases of the lumbar spine.

### Patient Groups

Patients of similar age, weight, and severity of the imaging‐based diagnosis and symptoms were divided into three groups according to the different treatment methods. Their baseline data showed no significant differences. Patients (*n* = 29) who had undergone the Wiltse TLIF operation were assigned to the Wiltse group. Patients (*n* = 28) who had undergone the MIS‐TLIF operation were categorized as the MIS‐TLIF group. Patients (*n* = 30) who had undergone the traditional PLIF operation were assigned to the PLIF group. All surgeries were performed by the same surgeon (Wei Zhang).

### Description of the New Retractor

**Fig. 1:**
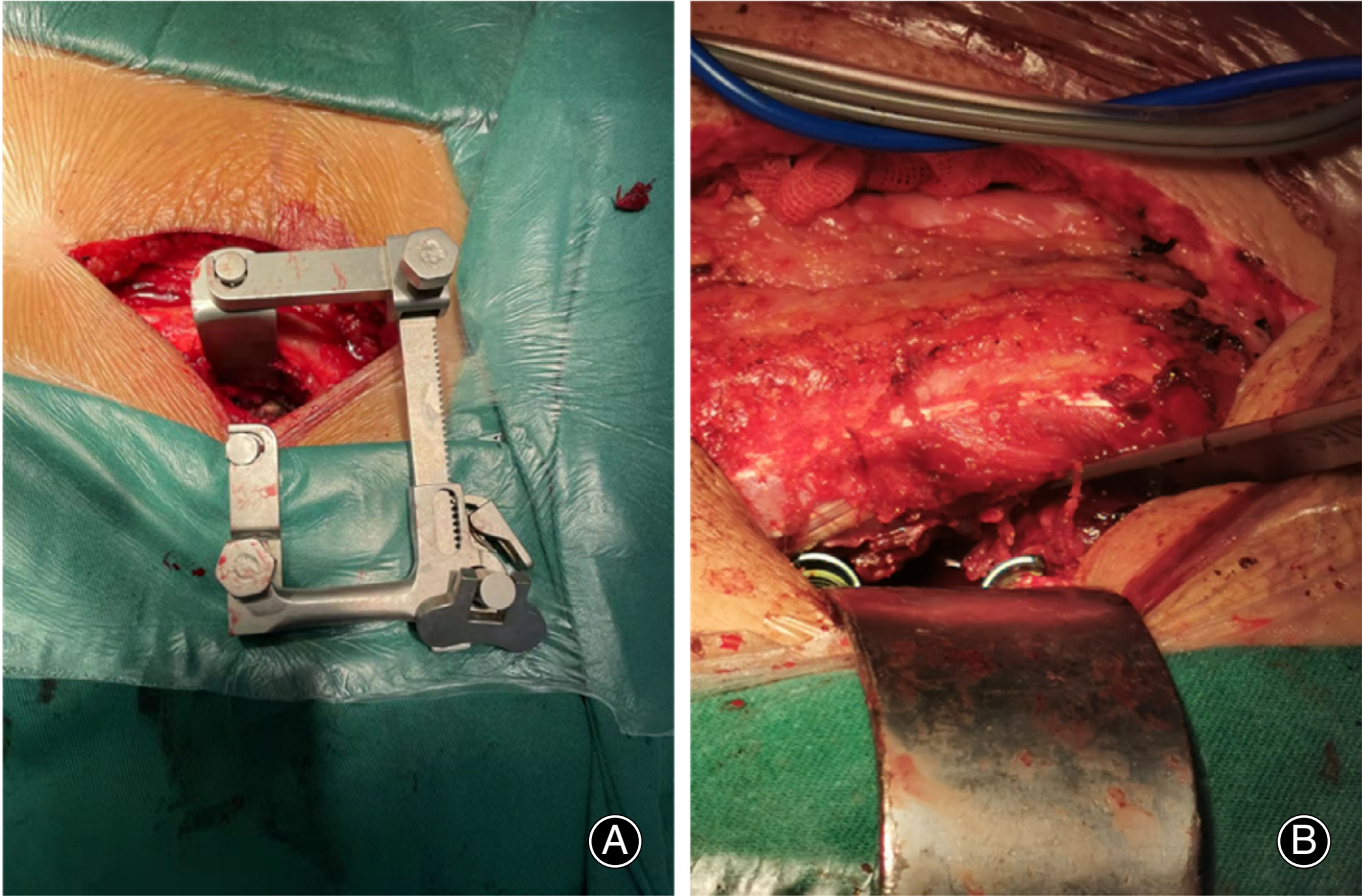
(A) Wiltse approach TLIF assisted by the new‐type retractor, the anatomical structure can be identified easily and decompression and fusion become more intuitive and convenient. (B) A traditional Wiltse approach TLIF surgery. It requires more energy from the assistant and is still hard to expose especially in obese or muscular patients

**Fig. 2:**
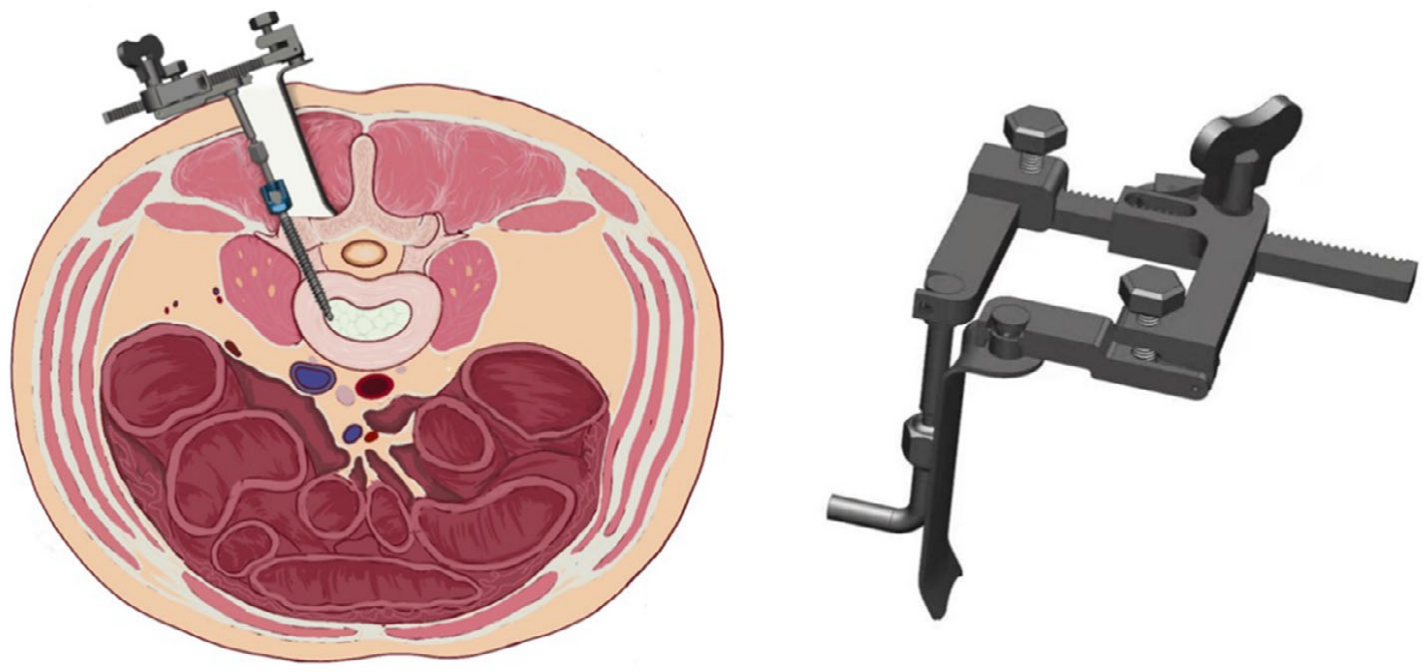
The new‐type retractor for Wiltse approach TLIF. After pedicle screw implantation, the retractor can be fixed on the pedicle screw. The rod attached to the pedicle screw pulls the muscle laterally, and the separation blade pulls the muscle medially. The distance and degree of retraction can be adjusted by rotating the knob

The retractor has a short metal rod at one end and a movable blade structure at the other end. The short metal rod end can be locked into the pedicle screw by nut fixation. The retractor can then be fixed on the pedicle screw. Figures 1A and 2 show the Wiltse TLIF assisted by this new retractor; the anatomical structure can be identified easily, and decompression and fusion become more intuitive and convenient. Figure 1B shows a traditional Wiltse TLIF surgery, which requires more effort from the assistant, with the exposure of anatomical structure being difficult, especially in obese or muscular patients.

### Surgical Procedures

#### Wiltse TLIF

First, after administering general anesthesia, the patients were placed in the prone position. Second, a Kirschner wire was used for positioning under C‐arm X‐ray fluoroscopic guidance to determine the stage of responsibility. Third, bilateral access was provided through an ‐8‐cm‐long midline skin incision. The lumbar dorsal fascia was incised longitudinally at 2.5–3 cm from the posterior midline^12^. The medial multifidus was separated from the lateral longissimus muscle using blunt dissection with fingers. Fourth, the junction of the facet joints and the transverse processes was identified, and the pedicle screw was installed. The retractor has a short metal rod at one end and a movable blade structure at the other end. The short metal rod ends were locked into the pedicle screw by nut fixation, and the retractor was fixed on the pedicle screw (Figures 1 and 2). Fifth, if the patient has bilateral disc herniation or spinal stenosis, the retractor can be fixed to the contralateral side in the same manner, followed by decompression of the contralateral side. The diseased intervertebral disc was resected, the cartilage endplate was scraped, and bone fragments and intervertebral fusion cage were implanted into the intervertebral space. Sixth, the rod system was installed. Seventh, one drainage tube was placed beside the incision.

#### Expandable Tubular Retractor‐Assisted MIS‐TLIF

First, after administering general anesthesia, the patients were placed in the prone position. The projection position of the adjacent pedicle of the diseased segment was identified and marked using the C‐arm X‐ray machine. Second, guided by fluoroscopy, a percutaneous needle was used to locate the outer edge of each pedicle at the respective marking points. Four ~1.5‐cm‐long transverse incisions were made by centering each puncture point. The lumbar dorsal fascia was incised longitudinally. The space between the medial multifidus and longissimus muscles was investigated and the articular facet joint was explored along this gap. Third, after puncturing the articular process with a puncture needle and inserting the guide wire along each puncture needle catheter, an expandable tubular retractor was inserted along the guide wire. The retractor was placed on the medial side of the articular process and on the upper margin of the intervertebral space in the responsible stage. Fourth, after full decompression of the spinal and nerve root canals, the diseased intervertebral disc was resected, the cartilage endplate was scraped, and bone fragments and intervertebral fusion cage were implanted into the intervertebral space. If there were contralateral symptoms, the channel was adjusted along the spinous process base to complete the contralateral nerve root canal decompression and enlarge the contralateral nerve root canal and the central vertebral canal^5^. Fifth, after decompression and bone grafting, the retractor was removed from the working channel and hollow pedicle screws inserted into each pedicle along the guide wire. After the fluoroscopic position was satisfactorily confirmed, the pedicle screw rod system was installed. Sixth, a drainage tube was placed through the incision on the decompression side.

#### Traditional PLIF

First, after administering general anesthesia, the patients were placed in the prone position. Second, guided by a C‐arm X‐ray machine, a Kirschner wire was used to locate and determine the responsibility stage. Third, a ‐10‐cm‐long posterior midline incision was made. After the lumbar fascia was incised, the lateral paravertebral muscles along the spinous process were stripped to the bilateral facet joints. Fourth, the pedicle screw rod system was installed. Fifth, after full decompression of the contralateral spinal and nerve root canals, the diseased intervertebral disc was resected, the cartilage endplate was scraped, and bone fragments and intervertebral fusion cage were implanted into the intervertebral space. Sixth, the pedicle screw rod system was installed. Seventh, one drainage tube was placed next to the incision.

### Outcome Measures

#### Perioperative Indicators

Perioperative indicators included intraoperative blood loss, postoperative drainage volume, operation time, fluoroscopy time, and bedridden time.

#### Indicators of Muscle Injury

Creatine kinase (CK)

The serum CK concentration of the patients were measured using a spectrometric enzyme coupling method before surgery and 1, 4, and 7 days after surgery to evaluate the intensity of muscle injury. CK catalyzes the reaction of creatine with adenosine triphosphate (ATP) to yield phosphocreatine and adenosine diphosphate (ADP). Serum CK concentrations have been used to investigate skeletal muscle injury caused by lumbar surgery. Normal CK levels are considered to be in the range of 20–200 IU/L.

#### Visual Analog Scale (VAS)

Lower back pain and lower limb pain scores were evaluated at 3 days and 3, 6, and 12 months after surgery. The visual analog scale (VAS) is commonly used for the measurement of pain. It is a self‐reported scale consisting of a horizontal or vertical line, usually 10 cm long (100 mm) with anchor descriptors such as “no pain” in the pain context and “worst pain imaginable” in the pain status. An introductory question (with or without a time recall period) asks the patient to tick the line on the point that best refers to his/her pain. VAS is feasible for clinical research and practice.

#### Oswestry Disability Index (ODI)

In this study, patients were surveyed about nine aspects, omitting sexual activity from the ODI score system. The ODI was evaluated before surgery and 3, 6, and 12 months after surgery. The ODI is a set of principal condition‐specific outcome measures used in the management of spinal disorders to assess a patient's progress in routine clinical practice. The ODI score system includes 10 sections: pain intensity, personal care, lifting, walking, sitting, standing, sleeping, sex life, social life, and traveling. The total score is five for each section of six statements. Intervening statements are scored according to rank. The highest score is considered if more than one box is marked in each section. If all 10 sections are completed, the score is calculated as follows: total score out of total possible score × 100. If one section is missed (or not applicable), the score is calculated as: (total score/[5 × number of questions answered]) × 100%. Scores in the 0%–20% range indicate mild dysfunction, 21%–40% moderate dysfunction, 41%–60% severe dysfunction, and 61%–80% disability. Cases with scores of 81%–100% were either long‐term bedridden patients or were exaggerating the impact of pain on their life.

#### Japanese Orthopaedic Association (JOA) Score for Low Back Pain

The JOA scores for LBP were evaluated before surgery and 3, 6, and 12 months after surgery. The JOA score for back pain was established for evaluating LBP and/or lumbar spinal diseases and has been used to estimate the severity of LBP or clinical outcomes. The JOA score consists of four subscales: subjective symptoms, clinical signs, activities of daily living (ADL), and urinary bladder function, providing clinicians with significant information. The total possible JOA score is 29.

#### Evaluation of Intervertebral Fusion

In this study, two methods were used to evaluate intervertebral fusion at the last follow‐up: lumbar radiography of hyperextension and hyperflexion positions and lumbar CT in the extension position. The radiographs were interpreted as showing incomplete union if there was mobility of more than 3°, a remaining clear zone, or no definite bone connection^13^. A lumbar CT scan in hyperextension was considered incomplete fusion if there was a gas pattern, a remaining clear zone, or no definite bone connection^14^, ^15^, ^16^.

### Evaluation of Muscle Atrophy and Fatty Infiltration

**Fig. 3:**
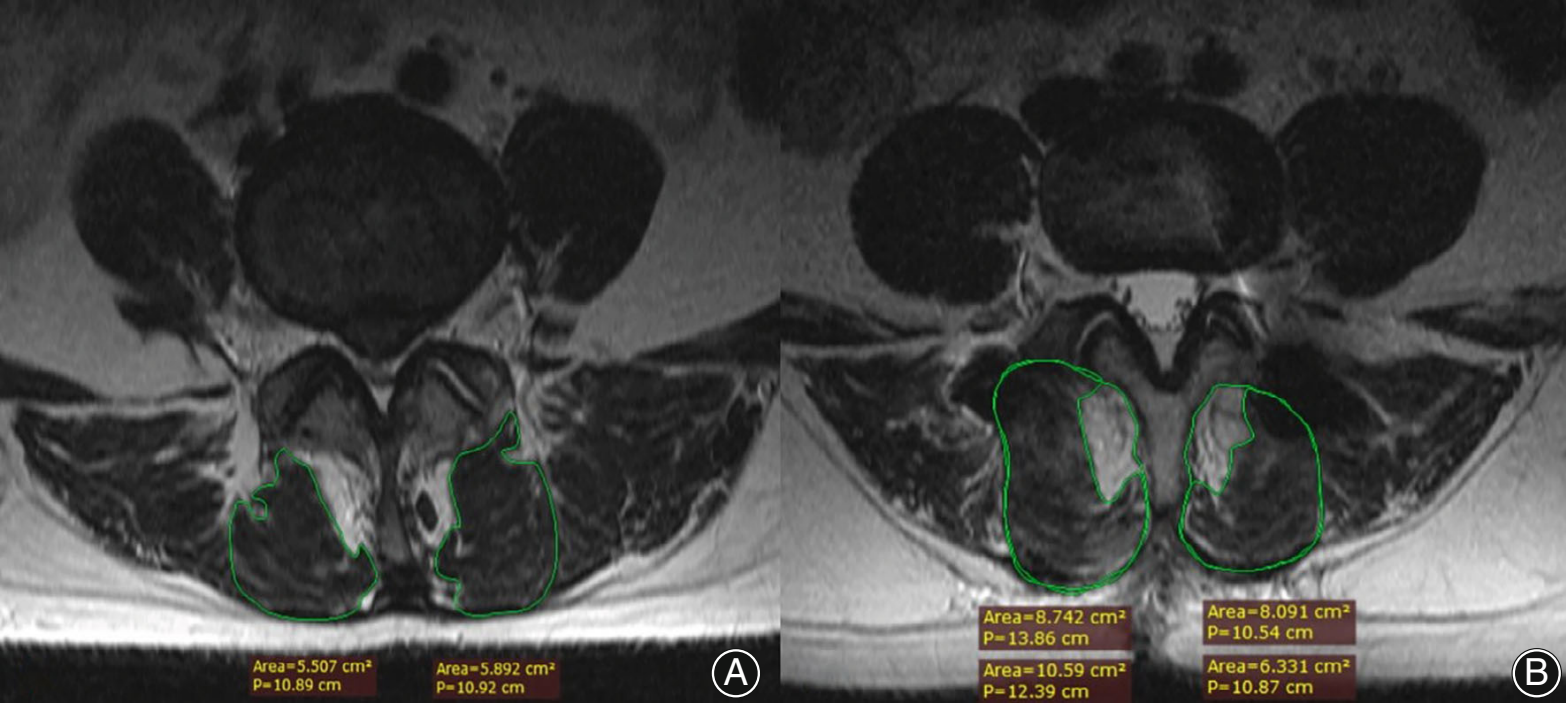
(A) Preoperative axial T2‐weighted MRI image demonstrates lean cross‐sectional area measurements. (B) Postoperative axial T2‐weighted MRI image demonstrates total and lean cross‐sectional measurements

Lumbar MRI was used to evaluate the multifidus atrophy and fatty infiltration. The results, including the total cross‐sectional area (CSA), lean CSA, and ratio of lean‐to‐total CSA, were evaluated before surgery and at the last follow‐up after surgery according to the following methods. The CSA was measured at the operative level using the axial T2‐weighted sequences obtained with the RadiAnt DICOM Viewer software (Medixant. RadiAnt DICOM Viewer, version 2020.2. July 19, 2020. https://www.radiantviewer.com.). To identify lean CSA, the region of interest (ROI) was drawn around the multifidus on both sides of the spinous process, excluding nearby fat, bone, and other tissues (Figure 3A). The following formula was used: ratio of multifidus atrophy = (preoperative CSA – postoperative CSA)/preoperative CSA. The ratio of lean‐to‐total CSA (Figure 3B) was utilized as an additional measurement of fatty infiltration, as described in papers on rotator cuffs: ratio of lean‐to‐total CSA = lean CSA /total CSA^17^, ^18^.

### Statistical Analysis

SPSS 20.0.0 statistical software was used for data analysis. For measurement data, statistical analysis was performed using one‐way ANOVA if the data were normally distributed and satisfied the homogeneity test of variance. Otherwise, the nonparametric test of rank conversion was used. Counting data were compared using the chi‐square test. The test level was 0.05 on both sides, and *p* ≤ 0.05 was considered statistically significant.

## Clinical Results

### Follow‐up Time

Patients were followed up at 3 months, 6 months, and 12 months after surgery. The mean follow‐up time was 12.14 ± 2.78 months in the Wiltse group, 13.57 ± 2.60 months in the MIS‐TLIF group, and 12.73 ± 2.80 months in the PLIF group, with no significant difference among the three groups (*p* > 0.05).

### Demographics

There were no significant differences in age, sex, the distribution of the surgical segment, preoperative VAS scores, preoperative ODI scores, and preoperative JOA scores (Table 1).

**TABLE 1:** Comparison of demographics among Wiltse, MIS‐TLIF, and PLIF group

	Wiltse	MIS‐TLIF	PLIF	*F*	*p*
Gender				0.445	0.642
Male	13	12	16		
Female	16	16	14		
Age (years)	52.38 ± 10.25	49.54 ± 10.78	51.17 ± 9.81	0.594	0.580
Surgical segment					
L_4‐5_	17	13	14		
L_5_‐S_1_	12	15	16		
Preoperative					
Low back pain VAS	3.17 ± 2.42	2.68 ± 2.41	2.80 ± 2.17	0.350	0.706
Lower extremity pain VAS	5.34 ± 2.21	4.96 ± 2.25	4.80 ± 2.12	0.476	0.623
ODI (%)	63.46 ± 11.36	66.35 ± 11.71	64.22 ± 12.21	0.422	0.657
JOA score	12.21 ± 5.34	11.71 ± 4.96	11.70 ± 5.21	0.090	0.914

Abbreviations: Wiltse, Wiltse approach transforaminal lumbar interbody fusion; MIS‐TLIF, minimally invasive transforaminal lumbar interbody fusion; PLIF, posterior lumbar interbody fusion.

### Perioperative Metrics

Preoperative metrics can be viewed in Table 2. The three groups of surgery were completed by the same surgeon, and there was no change in surgical method during the operations. Intraoperative blood loss (*F* = 62.628, *p* < 0.001) and postoperative drainage volume (*F* = 72.048，*p* < 0.001) were significantly different for the three groups. They were significantly lower for the MIS‐TLIF and Wiltse groups than the PLIF group and lower for the MIS‐TLIF group than the Wiltse group.

**TABLE 2:** Comparison of perioperative metrics between Wiltse, MIS‐TLIF, and PLIF group

	Wiltse	MIS‐TLIF	PLIF	*F*	*p*
Intraoperative bleeding (ml)	241.38 ± 98.26a	164.29 ± 65.06b	428.33 ± 65.06c	62.628	<0.001
Postoperative drainage (ml)	127.24 ± 41.99a	53.93 ± 25.44b	182.67 ± 50.37c	72.048	<0.001
Operation time (min)	98.97 ± 24.98a	169.11 ± 29.38b	94.67 ± 25.43a	70.289	<0.001
Fluoroscopy time (s)	22.41 ± 0.73a	87.61 ± 3.13b	22.41 ± 0.73a	403.114	<0.001
Bedridden time (day)	3.3 ± 0.48a	2.68 ± 0.71b	5.13 ± 0.90c	91.269	<0.001

*Note*: LSD method was used to compare the statistical differences between groups.

Abbreviations: Wiltse, Wiltse approach transforaminal lumbar interbody fusion; MIS‐TLIF, minimally invasive transforaminal lumbar interbody fusion; PLIF, posterior lumbar interbody fusion.

The operative time showed significant differences among the three groups. The operative time of the MIS‐TLIF group (169.11 ± 29.38 min) was significantly longer than that of the Wiltse group (94.67 ± 25.43 min) and PLIF group (98.97 ± 24.98 min). The fluoroscopy time of the MIS‐TLIF group (87.61 ± 3.13 s) was significantly longer than that of the Wiltse group (22.41 ± 0.73 s) and PLIF group (22.41 ± 0.73 s).

Postoperative bedridden time differed significantly among the three groups (χ^2^ = 62.289，*p* < 0.001). The postoperative bedridden time of the MIS‐TLIF group (2.68 ± 0.71 days) and the Wiltse group (3.3 ± 0.48 days) was significantly shorter than that of the open approach group (5.13 ± 0.90 days). The postoperative bedridden time of the MIS‐TLIF group was shorter than that of the Wiltse group.

### Evaluation of Paravertebral Muscle Injury

**Fig. 4:**
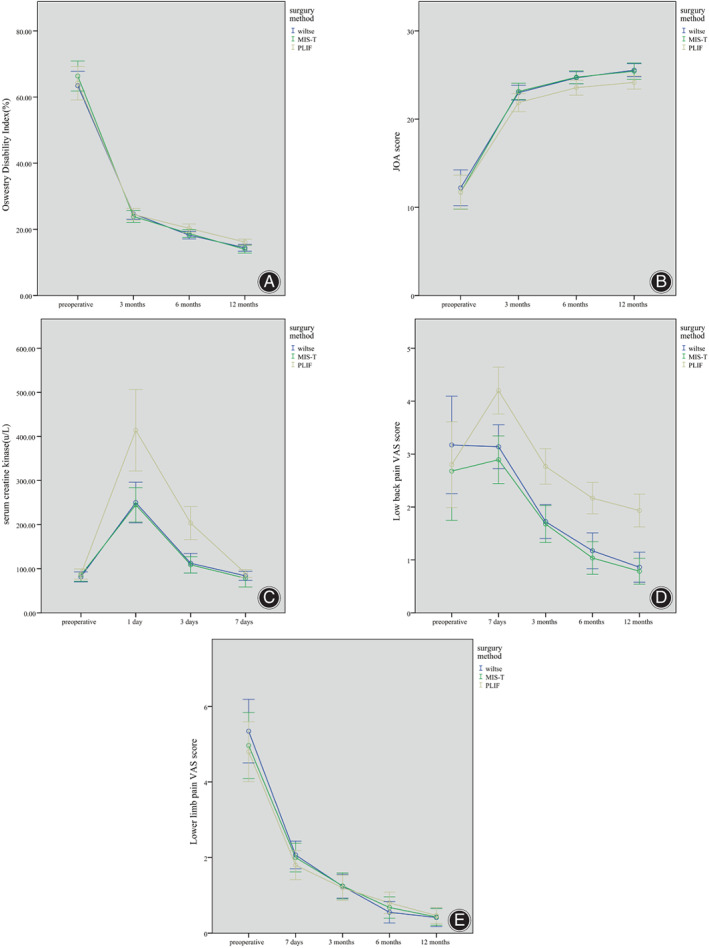
(A) ODI of Wiltse, MIS‐TLIF group at 6 and 12 months after surgery were lower than PLIF. (B) JOA score of Wiltse, MIS‐TLIF group at 6 and 12 months after surgery were higher than PLIF group. (C) Serum creatine kinase level of Wiltse, MIS‐TLIF group on 1 and 3 day(s) after surgery were significantly lower than PLIF group. (D) Low back pain VAS score of Wiltse, MIS‐TLIF group at 7 days, 3, 6, 12 months after surgery were significantly lower than PLIF group. (E) lower limb pain VAS score of Wiltse, MIS‐TLIF, and PLIF group showed no statistical difference

There were no significant differences in the CK concentration at preoperative (*F* = 0.307, *p* = 0.736) and 7 days after surgery (*F* = 0.670, *p* = 0.515) among the three groups. There were significant differences in CK 1 day (*F* = 9.331, *p* < 0.001) and 3 days (*F* = 15.967, *p* < 0.001) after surgery. The CK concentration of the Wiltse and MIS‐TLIF groups were significantly lower than those of the PLIF group. However, there were no significant differences between the MIS‐TLIF and Wiltse groups in the CK concentration 1 and 3 days after surgery (*p* = 0.907, *p* = 0.860) (Table 3 & Figure 4C).

**TABLE 3:** CK(u/L) level of Wiltse, MIS‐TLIF, and PLIF group

	Preoperative	1 day	3 days	7 days	*F*	*p*
Wiltse	81.41 ± 29.51a	250.07 ± 120.17b	112.31 ± 58.52c	83.79 ± 27.65a	**19.285** *	<**0.001** *
MIS‐TLIF	85.54 ± 35.95a	244.75 ± 100.26b	108.89 ± 47.76c	78.39 ± 51.04a	**21.012** *	<**0.001** *
PLIF	87.93 ± 32.00a	414.10 ± 247.49b	203.60 ± 99.97a	89.30 ± 23.76a	**15.840** *	<**0.001** *
*F* value	0.307^&^	9.331^&^	15.967^&^	0.670^&^	**–**	**–**
*p* value	0.736^&^	<**0.001** ^&^	<**0.001** ^&^	0.515^&^	**–**	**–**

*Note*: Bold indicates statistically significant values. Multiple comparisons of CK at different time points using the Bonferroni *post hoc* test, and at least one identical subscript letter denoted no significant difference from each other at the 0.05 level.

Abbreviations: Wiltse, Wiltse approach transforaminal lumbar interbody fusion; MIS‐TLIF, minimally invasive transforaminal lumbar interbody fusion; PLIF, posterior lumbar interbody fusion.

^&^ One‐way analysis of variance.

* Repeated measurement analysis of variance.

### Low Back Pain VAS Score and Lower Limb Pain VAS Score

There were no significant differences in LBP VAS scores among the three groups before the operations (*F* = 0.350, *p* = 0.706). The back pain VAS scores 7 days, 3 months, 6 months, and 12 months after surgery showed significant differences among all three groups. The PLIF group back pain VAS score was higher than those of the Wiltse and MIS‐TLIF groups, whereas there was no significant difference in the scores between the MIS‐TLIF and Wiltse groups. The back pain VAS scores were compared at different times within the groups using analysis of variance for single‐factor repeated measurements. The PLIF group back pain VAS score 7 days after the surgery (1.4 ± 0. 388) was higher than the pre‐operation score, whereas the MIS‐TLIF and Wiltse groups showed no significant increase in the scores for this time point. The back pain VAS scores decreased gradually for all three groups at 3 months, 6 months, and 12 months after the surgery.

There was no significant difference in the lower limb pain VAS scores among the three groups before surgery (*F* = 0.476, *p* = 0.623) and at 7 days (*F* = 0.586, *p* = 0.559), 3 months (*F* = 0.028, *p* = 0.973), 6 months (*F* = 0.828, *p* = 0.440), and 12 months after the surgery (*F* = 0.063, *p* = 0.939) (Table 4 & Figure 4D and E).

**TABLE 4:** VAS score of Wiltse, MIS‐TLIF, and PLIF group

VAS	Preoperative	7 days	3 months	6 months	12 months	*F*	*p*
*Low back pain*							
Wiltse	3.17 ± 2.42a	3.14 ± 1.09a	1.72 ± 0.84c	1.17 ± 0.89d	0.86 ± 0.74e	**32.889** *	<**0.001** *
MIS‐TLIF	2.68 ± 2.41a	2.89 ± 1.17a	1.68 ± 0.91c	1.04 ± 0.79d	0.79 ± 0.63e	**38.801** *	<**0.001** *
PLIF	2.80 ± 2.17a	4.20 ± 1.19b	2.77 ± 0.90a	2.17 ± 0.79c	1.93 ± 0.83d	**39.072** *	<**0.001** *
*F* value	0.350	**10.729**	**14.363**	**16.435**	**22.124**	‐	‐
*p* value	0.706^&^	<**0.001** ^&^	<**0.001** ^&^	<**0.001** ^&^	<**0.001** ^&^	‐	‐
*Lower limb pain*							
Wiltse	5.34 ± 2.21a	2.07 ± 0.96b	1.24 ± 0.83c	0.55 ± 0.74d	0.41 ± 0.63e	**36.783** *	<**0.001** *
MIS‐TLIF	4.96 ± 2.25a	2.00 ± 0.98b	1.25 ± 0.89c	0.68 ± 0.72d	0.43 ± 0.57e	**31.628** *	<**0.001** *
PLIF	4.80 ± 2.12a	1.80 ± 1.03b	1.20 ± 0.89c	0.80 ± 0.76d	0.47 ± 0.57e	**33.415** *	<**0.001** *
*F* value	0.476	0.586	0.028	0.828	0.063	‐	‐
*p* value	0.623^&^	0.559^&^	0.973^&^	0.440^&^	0.939^&^	‐	‐

*Note*: Bold indicates statistically significant values. Multiple comparisons of each variable at different time points using the Bonferroni *post hoc* test, and at least one identical subscript letter denoted no significant difference from each other at the 0.05 level.

Abbreviations: Wiltse, Wiltse approach transforaminal lumbar interbody fusion; MIS‐TLIF, minimally invasive transforaminal lumbar interbody fusion; PLIF, posterior lumbar interbody fusion.

^&^ One‐way analysis of variance.

* Repeated measurement analysis of variance.

### Oswestry Disability Index (ODI)

There was no significant difference in the ODI among all three groups pre‐operation (*F* = 0.422, *p* = 0.657) and 3 months after surgery (*F* = 0.230, *p* = 0.795). However, the ODIs were significantly different 6 months (*F* = 3.282, *p* = 0.042) and 12 months after surgery (*F* = 5.316, *p* = 0.007). The ODI of the PLIF group was higher than those of the Wiltse and MIS‐TLIF groups. However, there was no significant difference in the ODI between the Wiltse and MIS‐TLIF groups. ODI values were compared at different times within the groups using analysis of variance for single‐factor repeated measurements. The ODI of all three groups decreased gradually at 3, 6, and 12 months after surgery (Table 5 and Figure 4A).

**TABLE 5:** ODI and JOA score of Wiltse, MIS‐TLIF, and PLIF group

	Preoperative	3 months	6 months	12 months	*F*	*p*
ODI (%)						
Wiltse	63.46 ± 11.36a	24.67 ± 4.34b	18.24 ± 2.99c	14.41 ± 2.70d	**247.685** *	<**0.001** *
MIS‐TLIF	66.35 ± 11.71a	23.89 ± 4.67b	18.73 ± 3.05c	13.96 ± 3.02d	**218.918** *	<**0.001** *
PLIF	64.22 ± 13.63a	24.52 ± 4.83b	20.30 ± 3.58c	16.15 ± 2.40d	**173.905** *	<**0.001** *
*F* value	0.422^&^	0.230^&^	**3.282** ^&^	**5.316** ^&^	‐	‐
*p* value	0.657^&^	0.795^&^	<**0.042** ^&^	<**0.007** ^&^	‐	‐
JOA score						
Wiltse	12.21 ± 5.34a	23.00 ± 2.20b	24.69 ± 1.82c	25.55 ± 1.94d	**85.793** *	<**0.001** *
MIS‐TLIF	11.71 ± 4.96a	23.14 ± 2.40b	24.75 ± 1.88c	25.43 ± 2.38d	**82.896** *	<**0.001** *
PLIF	11.70 ± 5.21a	21.87 ± 2.73b	23.57 ± 2.29c	24.17 ± 2.02d	**86.844** *	<**0.001** *
*F* value	0.090^&^	2.383^&^	**3.242** ^&^	**3.874** ^&^	–	–
*p* value	0.914^&^	0.098^&^	**0.044** ^&^	**0.025** ^&^	–	–

*Note*: Bold indicates statistically significant values. Multiple comparisons of each variable at different time points using the Bonferroni *post hoc* test, and at least one identical subscript letter denoted no significant difference from each other at the 0.05 level.

Abbreviations: Wiltse, Wiltse approach transforaminal lumbar interbody fusion; MIS‐TLIF, minimally invasive transforaminal lumbar interbody fusion; PLIF, posterior lumbar interbody fusion.

^&^ One‐way analysis of variance.

* Repeated measurement analysis of variance.

### JOA Score

There were no significant differences in the preoperative JOA score (*F* = 0.09, *p* = 0.914) and the JOA scores 3 months after the surgery (*F* = 2.383, *p* = 0.098) among all three groups. However, there were significant differences 6 months (*F* = 3.234, *p* = 0.044) and 12 months (*F* = 3.874, *p* = 0.025) after the surgery. The JOA score of the PLIF group was lower than those of the Wiltse and MIS‐TLIF groups 6 months and 12 months after surgery. There were no significant differences in the JOA scores between the Wiltse and MIS‐TLIF groups at 6 months (*p* = 0.091) and 1 year (*p* = 0.827) after surgery. The JOA scores were compared at different times within the groups using analysis of variance for single‐factor repeated measurements. The JOA scores increased gradually at 3, 6, and 12 months after the surgery for all three groups (Table 5 and Figure 4B).

### Intervertebral Fusion Rate

**Fig. 5:**
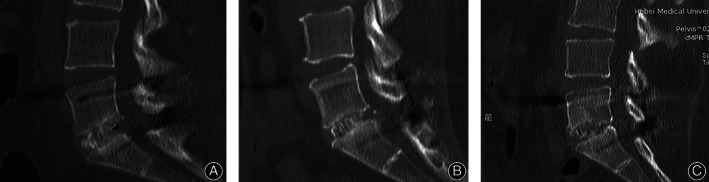
Wiltse, MIS‐TLIF, and PLIF group showed definite bone connection. (A) 12 months CT of a Wiltse approach TLIF patient. (B) 14 months CT of a MIS‐TLIF patient. (C) 36 months CT of a PLIF patient

The intervertebral fusion rate was 89.7% in the Wiltse group, 92.9% in the MIS‐TLIF group, and 93.3% in the PLIF group (Figure 5). There was no significant difference in the follow‐up time (*F* = 1.975, *p* = 0.145) and intervertebral fusion rate (χ^2^ = 0.315, *p* = 0.854) among all three groups.

### Multifidus Atrophy and Fatty Infiltration

**Fig. 6:**
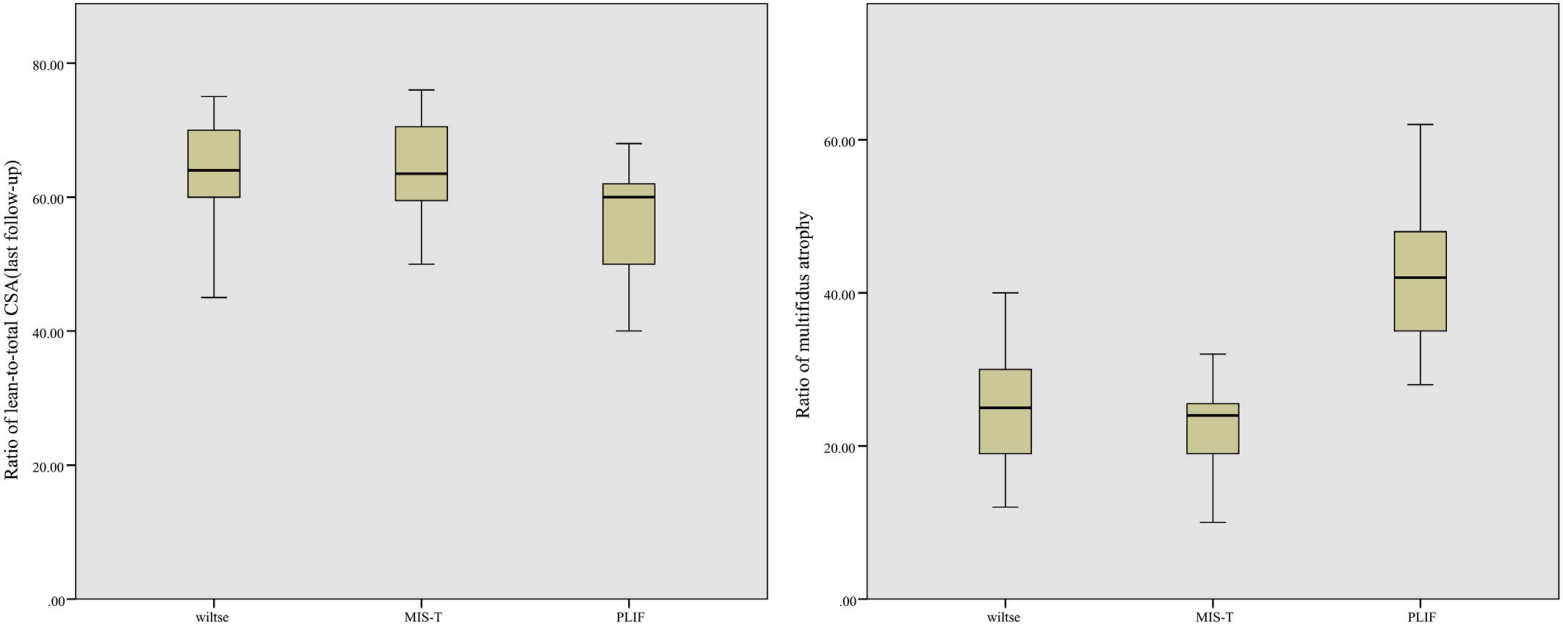
Ratio of lean‐to‐total CSA:PLIF group (56.60% ± 7.52%) was lower than in Wiltse group (63.34% ± 7.74%) and MIS‐TLIF group (64.03% ± 7.19%). Ratio of multifidus atrophy: PLIF group (41.70% ± 8.84%) was significantly higher than that in the Wiltse group (24.13% ± 6.82%) and the MIS‐TILF group (22.35% ± 5.03%)

**Fig. 7:**
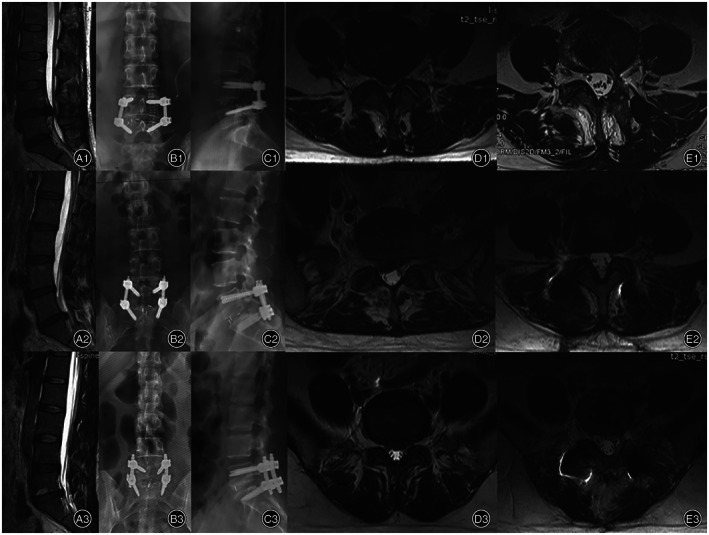
(A1–E1) One case of Wiltse approach TLIF patient. (A2–E2) One case of MIS‐TLIF patient, (A3–E3) One case of PLIF patient. (A1) MRI before surgery showed disc herniation in L4‐L5 segment. (B1 and C1) Postoperative anterior and lateral DR. (D1) Axial T2 MRI show disc herniation and multifidus before surgery. (E1) Axial T2 MRI show surgery level multifidus at last follow‐up after surgery with no significant atrophy and fatty infiltration. (A2) MRI before surgery showed disc herniation in L5‐S1 segment. (B2 and C2) postoperative anterior and lateral DR. (D2) Axial T2 MRI show disc herniation and multifidus before surgery. (E2) Axial T2 MRI show surgery level multifidus at last follow‐up after surgery with no significant atrophy and fatty infiltration. (A3) MRI before surgery showed disc herniation in L5‐S1 segment. (B3 and C3) Postoperative anterior and lateral DR. (D3) Axial T2 MRI show disc herniation and multifidus before surgery. (E3) Axial T2 MRI show surgery level multifidus at last follow‐up after surgery with significant atrophy and fatty infiltration.

The ratio of multifidus atrophy in the PLIF group (41.70% ± 8.84%) was significantly higher than that in the Wiltse group (24.13% ± 6.82%) and the MIS‐TLIF group (22.35% ± 5.03%). However, there was no significant difference between the Wiltse and MIS‐TLIF groups (*p* = 0.348) (Table 6 and Figure 6). There was no significant difference in the ratio of lean‐to‐total CSA among the three groups before surgery (*F* = 0.749, *p* = 0.476). However, there were significant differences after surgery (*F* = 8.852, *p* < 0.001), with the ratio being lower in the PLIF group (56.60 % ± 7.52%) than in the Wiltse group (63.34% ± 7.74%) and the MIS‐TLIF group (64.03%± 7.19%) (Figure 7). There was no significant difference between the ratios for the Wiltse and MIS‐TLIF groups (*p* = 0.729).

**TABLE 6:** Comparison of interbody fusion, multifidus atrophy, and fatty infiltration among three groups

	Wiltse	MIS‐TLIF	PLIF	*F*	*p*
Follow‐up time (months)					
CT	12.14 ± 2.78	13.57 ± 2.60	12.73 ± 2.80	1.979	0.145
MRI	11.84 ± 3.07	12.25 ± 2.70	13.16 ± 3.0	0.650	0.528
Interbody fusion rate (%)	89.7	92.9	93.3	0.153	0.854
Ratio of multifidus atrophy (%)	24.13 ± 6.82a	22.35 ± 5.03a	41.70 ± 8.84b	**66.438**	<**0.001**
Ratio of lean‐to‐total CSA					
Pre‐operation (%)	69.28 ± 8.11	70.64 ± 7.92	68.17 ± 7.09	0.749	0.476
Last follow‐up (%)	63.34 ± 7.74a	64.03 ± 7.19a	56.60 ± 7.52b	**8.852**	<0**.001**

*Note*: Bold indicates statistically significant values. LSD method was used to compare the statistical differences between groups and at least one identical subscript letter denoted no significant difference from each other at the 0.05 level.

Abbreviations: Wiltse, Wiltse approach transforaminal lumbar interbody fusion; MIS‐TLIF, minimally invasive transforaminal lumbar interbody fusion; PLIF, posterior lumbar interbody fusion.

### Intraoperative Results

The Wiltse approach can significantly reduce the amount of bleeding during surgical exposure. Muscle spaces are easier to find and separate in the cranial side, especially at the L4‐5 segment and above. However, the muscle space is more external to the L5‐S1 segment of the lumbar spine, and the boundary is more blurred. In addition, the pedicle screw implantation requires a larger abduction angle, which is more likely to cause muscle damage. In the blunt separation of the multifidus and longissimus muscles, it is easier to separate the muscle spaces by proceeding from the cranial to the caudal side. Therefore, relatively speaking, the L5‐S1 segment muscle damage is relatively higher in the Wiltse‐approach surgery, especially in robust and muscular patients. During suturing, the fascia layer is thinner than the midline, which requires careful suturing to avoid muscle hernia, which may lead to postoperative LBP and discomfort.

### Surgical Complications

There were two cases of durotomy in the PLIF group, but no severe complication, such as central nervous system infection, after complete suture and duraplasty. No dural rupture was found in the Wiltse and MIS‐TLIF groups. In the PLIF group, there were two cases of incision fat liquefaction, which healed after dressing change and debridement. In contrast, no fat liquefaction occurred in the Wiltse and MIS‐TLIF groups. In the MIS‐TLIF group, one case of skin edge necrosis was found, which recovered after the excision of the skin edge suture. No instrumental failure and loosening was found in any group.

## Discussion

### New Retractor for Wiltse TLIF: A Simple, Convenient Device to Improve Exposure

The Wiltse approach has traditionally used a laminectomy retractor to expose the surgical field. It is fixed by the assistant on the lateral margin of the facet joint, pulling the longissimus muscle to the lateral margin. In muscular or obese patients, the multifidus muscle may also occlude the surgical field and affect surgical procedures. To further increase the surgical convenience of this approach, we independently designed a new muscle retractor that can be fixed on the pedicle screw. After the pedicle screw is inserted, the lateral part of the retractor can be fixed to the pedicle screw and locked by tightening the nut, so that this part can pull the longissimus muscle laterally. The medial part of the retractor is an adjustable metal blade that pulls the multifidus muscle medially; the degree of pull can be adjusted using the knob.

The advantages of this retractor are as follows: (i) a greater intraoperative field is obtained by pulling through the multifidus muscle medially; (ii) the fixation method is simple and the operation time shortened; (iii) there is no need for an assistant to retract continuously, making the operation more convenient. The primary application of this new retractor for the Wiltse approach has achieved a good clinical effect. The Wiltse approach is minimally invasive and causes little damage to the normal structure. It also has the advantages of a large operative field and convenience associated with open surgery. The Wiltse approach is widely used in the treatment of thoracolumbar fractures and lumbar degenerative disease, allowing the operator to directly reach the facet joints. Therefore, it is convenient for thoracolumbar pedicle screw implantation, nerve decompression of foraminal and lateral foraminal areas, and interbody fusion. The Wiltse approach assisted by the new retractor has a wide range of application prospects, such as lumbar disc herniation, lumbar spinal stenosis, lumbar spondylolissin, discogenic LBP, and posterior screw implantation after XLIF/oblique lateral interbody fusion (OLIF).

### Wiltse TLIF and MIS‐TLIF: Two Minimally Invasive Ways Showing Similar Muscle Injury

The Wiltse TLIF and MIS‐TLIF methods have the advantages of a small incision, less multifidus injury, and quick postoperative recovery^19^. In this study, the Wiltse and MIS‐TLIF groups showed significantly reduced intraoperative blood loss, postoperative drainage volume, and postoperative bedridden time (Table 2), which is consistent with the findings of Lee *et al*.^20^. This is mainly due to the differences in the surgical approach. The posterior paraspinal muscles are mainly composed of the multifidus, longissimus, and iliocostalis. The lumbar multifidus is an important muscle for lumbar segmental instability. The medial branch of the dorsal rami innervates the fascicles of the multifidus attached to the spinous process and plays an important role in maintaining lumbar segmental stability^21^.

Extensive multifidus muscle stripping and retraction, as well as damage to the dorsal rami due to posterior lamina decompression of the posterior branches caused by traditional posterior surgery, will inevitably lead to increased intraoperative bleeding and more serious tissue damage. This will result in increased postoperative drainage volume and prolonged bedridden time, atrophy of the multifidus muscles, and chronic LBP. In contrast, the Wiltse TLIF and MIS‐TLIF reach the surgical site through the natural space between the multifidus and the longus muscles. Blunt muscle separation or tubular expansion can avoid direct cutting damage to the muscle tissue, which is more in accordance with the concept of minimally invasive surgery. This also helps avoid related complications caused by prolonged bed stay, such as deep vein thrombosis of the lower limbs and hypostatic pneumonia. Moreover, the intraoperative blood loss and postoperative drainage volume were less in the MIS‐TLIF group than in the Wiltse group. MIS‐TLIF has shown reduced surgery‐related bleeding to a greater extent.

Postoperative CK level can be used as an indicator of muscle injury^22^, ^23^, ^24^, ^25^. Zhang *et al*. showed a significantly lower CK level in the MIS‐TLIF group *vs* PLIF after surgery (*p* < 0.001)^26^. In this study, the CK levels of the Wiltse and MIS‐TLIF groups were significantly lower than those of the PLIF group on day 1 and day 3 after surgery. This confirmed the significantly lower degree of muscle injury during Wiltse TLIF and MIS‐TLIF than during PLIF surgery. No significant difference was observed in muscle injury between the Wiltse TLIF and MIS‐TLIF groups (Table 3 and Figure 4c). In Wiltse TLIF, the medial multifidus is separated from the lateral longissimus muscle using blunt dissection easily because there is a natural gap between the multifidus and the longissimus muscles. The exposure process will not cause distraction of muscle fibers or overstretching, which conforms to the concept of minimally invasive surgery. In MIS‐TLIF surgery, the surgeon must adjust the direction of the expandable tubular retractor to achieve adequate spinal decompression, especially contralateral decompression. In this process, the compression of the paravertebral muscles is relatively more severe. Thus, in addition to the long operation time, the degree of muscle damage caused by the MIS‐TLIF was significantly greater than that caused by Wiltse TLIF.

### New Retractor‐Assisted Wiltse TLIF More Effectively Shortened Operating and Fluoroscopy Time Compared to MIS‐TLIF

However, the operating and fluoroscopy times of MIS‐TLIF were significantly longer than those of Wiltse TLIF and traditional PLIF, which was consistent with the results reported by Phan *et al*.^24^. This could be attributed to minimally invasive exposures being limited to the area of surgical interest and certain key anatomic landmarks within this limited field of view. Thus, the surgeon needs a longer learning curve to become familiar with the anatomy of this region to safely perform the procedure without exposing structures that are not being surgically treated. To ensure the correct position of the working tubular and excise insertion of the pedicle screw, a long fluoroscopy time is inevitable. In our study, we found that the fluoroscopy time of MIS‐TLIF is three times longer than that of Wiltse TLIF, and the operation time is ‐60 min longer (Table 2).

Traditional Wiltse TLIF procedures use a conventional lumbar surgery retractor, which is not convenient and increases the difficulty of the operation. The new retractor used here can be fixed on the pedicle screw and facilitates surgical field exposure by adjusting the retractor blade and the junction of the facet joint. Thus, the transverse processes can be identified easily in Wiltse TLIF (Figures 1 and 2). The excision of hyperplastic ligamentum flavum and degenerative facet, the removal of the disc, internal fixation, and interbody fusion can be performed under intuitive and clear surgical vision. The operation is convenient, safe, and reliable, with sufficient decompression of the vertebral canal and reliable clinical efficacy. The Wiltse TLIF method is more suitable than MIS‐TLIF with respect to simplicity and convenience combined with the reduction of radiation to patients, doctors, and nurses.

### Wiltse TLIF and MIS‐TLIF More Effectively Alleviate Back Pain Compared to PLIF

Cheng *et al*. found that Wiltse VAS for back pain at 7 days and 3 months showed better results (*p* < 0.05), while VAS for leg pain showed better results at 3 months but no significant difference at 7 days *vs* the traditional approach^27^. In our study, back pain VAS scores were lower at 7 days, 3, 6, and 12 months after surgery in the MIS‐TLIF and Wiltse groups than in the PLIF group. There were no significant differences in the VAS scores for leg pain among the three groups. The differences in the findings of our study and the study by Cheng *et al*. may arise from the error in subjective assessment associated with VAS.

The PLIF group had a higher ODI but a lower JOA score at 6 and 12 months after the operation than the MIS‐TLIF and Wiltse groups. The difference in the ODI and JOA scores among the three groups was mainly related to postoperative, long‐term LBP resulting from the PLIF method. The lower extremity neurological symptoms of patients in each group were relieved, indicating that Wiltse TLIF and MIS‐TLIF could achieve satisfying decompression effects in parallel with the PLIF surgery. There was no significant difference in the intervertebral fusion rates among the three groups, which indicated that all three surgical methods could achieve the expected fusion results. In conclusion, Wiltse TLIF and MIS‐TLIF can effectively alleviate back pain on the basis of relieving neurological symptoms, and both achieve better clinical effects than PLIF.

### Wiltse TLIF and MIS‐TLIF More Effectively Reduce Paravertebral Muscle Injury Compared to PLIF

The long‐term effects on paravertebral muscle can be evaluated by MRI. The reduction in paravertebral CSA and infiltration of fat and connective tissue are mainly manifested as enhanced signals on T2‐weighted imaging^28^. Junhui *et al*. found that multifidus CSA at the final follow‐up MRI was significantly lower in the Wiltse group (CSA decreased by 7.6%) than the PLIF group (CSA decreased by 35.4%)^29^. In line with these findings by Junhui *et al*., our study also revealed that the ratio of multifidus atrophy was significantly lower in the Wiltse and MIS‐TLIF groups than in the PLIF group, while the difference between the Wiltse and MIS‐TLIF groups was not obvious (Table 6 and Figure 7).

The ratio of lean‐to‐total CSA helps compare the degree of fatty infiltration quantitatively. The results revealed that the ratio of lean‐to‐total CSA of the PLIF group was significantly lower than those of the Wiltse and MIS‐TLIF groups, while there was no significant difference in this ratio between the Wiltse and MIS‐TLIF groups (Table 6 and Figure 7). This confirmed, from an imaging perspective, that the Wiltse approach reduced the degree of multifidus muscle atrophy and retained more paravertebral muscle function compared with traditional PLIF surgery, which was helpful to maintain spinal stability. There was no significant difference between the Wiltse and MIS‐TLIF groups, indicating that these two surgical methods could achieve similar surgical effects in reducing paravertebral muscle injury.

### Intraoperative and Postoperative Complications

Complications of intraoperative dural rupture and incision fat liquefaction occurred in the PLIF group, while no similar complications occurred in the Wiltse and MIS‐TLIF groups. There was one case of skin edge necrosis in the MIS‐TLIF group but none in the other two groups. Both Wiltse TLIF and MIS‐TLIF methods involved small incisions and quick healing, which helped reduce the occurrence of postoperative complications. The occurrence of skin edge necrosis in the MIS‐TLIF group may be caused by a lack of surgical skill in the early phase of surgery, long operation time, and long compression time of fixed tube on the skin. No skin edge necrosis occurred when the operator was surgically competent, and the operation time was shorter.

### Limitations

This study has some limitations. First, this study is a retrospective, comparative study, and the number of cases is relatively small. Second, the follow‐up time of the patients is relatively short, and there is a lack of statistics and comparison of long‐term complications and surgical efficacy. Third, only patients with single‐level lumbar spine surgery were analyzed, and all of them were L4‐L5 and L5‐S1 disc level lesions. Further investigation of patients with multisegment and higher‐level disc degenerative diseases is necessary. Finally, the comparison of paravertebral muscle atrophy in this study was limited to MRI evaluation.

Relevant studies have shown that the results of pathology and electrophysiological assessment can further clarify the effects of different surgical procedures on paravertebral muscles from different perspectives^29^. The author believes that the advantages and disadvantages of MIS‐TLIF and Wiltse TLIF can be further explored through randomized, controlled trials; multicenter, long‐term follow‐up; the inclusion of more patients with multi‐segmental and intervertebral disc degenerative disease at a higher level; and the inclusion of more evaluation indicators, such as paravertebral muscle tissue pathology and paravertebral muscle electrophysiological analyses. The new retractor may increase the stress of the screw, especially in patients with osteoporosis. Hence, the position of the screw and the risk of screw offset and pedicle injury should be evaluated in future studies.

## Conclusion

MIS‐TLIF, Wiltse TLIF, and PLIF can achieve satisfactory surgical efficacy in single‐level degenerative diseases of the lumbar spine in well‐selected patients. MIS‐TLIF and Wiltse TLIF can significantly reduce bleeding, bedridden time, muscle injury, low back pain, paravertebral muscle atrophy, and fatty infiltration to a greater extent than PLIF surgery. Although MIS‐TLIF had less bleeding than the Wiltse approach, it showed a similar degree of muscle trauma to Wiltse TLIF. Considering the long learning curve and long operation and fluoroscopy times of MIS‐TLIF, the Wiltse TLIF method, assisted by the new retractor, is a more convenient and minimally invasive surgical method than MIS‐TLIF.
